# Privacy preserving data anonymization of spontaneous ADE reporting system dataset

**DOI:** 10.1186/s12911-016-0293-4

**Published:** 2016-07-18

**Authors:** Wen-Yang Lin, Duen-Chuan Yang, Jie-Teng Wang

**Affiliations:** Department of Computer Science and Information Engineering, National University of Kaohsiung, Nanzih District, Kaohsiung, 811 Taiwan, R.O.C

**Keywords:** Adverse drug reaction, ADR signal detection, Data anonymization, Privacy preserving data publishing, Spontaneous reporting system

## Abstract

**Background:**

To facilitate long-term safety surveillance of marketing drugs, many spontaneously reporting systems (SRSs) of ADR events have been established world-wide. Since the data collected by SRSs contain sensitive personal health information that should be protected to prevent the identification of individuals, it procures the issue of privacy preserving data publishing (PPDP), that is, how to sanitize (anonymize) raw data before publishing. Although much work has been done on PPDP, very few studies have focused on protecting privacy of SRS data and none of the anonymization methods is favorable for SRS datasets, due to which contain some characteristics such as rare events, multiple individual records, and multi-valued sensitive attributes.

**Methods:**

We propose a new privacy model called MS(*k*, θ^*^)-bounding for protecting published spontaneous ADE reporting data from privacy attacks. Our model has the flexibility of varying privacy thresholds, i.e., θ^*^, for different sensitive values and takes the characteristics of SRS data into consideration. We also propose an anonymization algorithm for sanitizing the raw data to meet the requirements specified through the proposed model. Our algorithm adopts a greedy-based clustering strategy to group the records into clusters, conforming to an innovative anonymization metric aiming to minimize the privacy risk as well as maintain the data utility for ADR detection. Empirical study was conducted using FAERS dataset from 2004Q1 to 2011Q4. We compared our model with four prevailing methods, including *k*-anonymity, (*X*, *Y*)-anonymity, Multi-sensitive *l*-diversity, and (*α*, *k*)-anonymity, evaluated via two measures, Danger Ratio (*DR*) and Information Loss (*IL*), and considered three different scenarios of threshold setting for θ^*^, including uniform setting, level-wise setting and frequency-based setting. We also conducted experiments to inspect the impact of anonymized data on the strengths of discovered ADR signals.

**Results:**

With all three different threshold settings for sensitive value, our method can successively prevent the disclosure of sensitive values (nearly all observed *DR*s are zeros) without sacrificing too much of data utility. With non-uniform threshold setting, level-wise or frequency-based, our MS(*k*, θ^*^)-bounding exhibits the best data utility and the least privacy risk among all the models. The experiments conducted on selected ADR signals from MedWatch show that only very small difference on signal strength (PRR or ROR) were observed. The results show that our method can effectively prevent the disclosure of patient sensitive information without sacrificing data utility for ADR signal detection.

**Conclusions:**

We propose a new privacy model for protecting SRS data that possess some characteristics overlooked by contemporary models and an anonymization algorithm to sanitize SRS data in accordance with the proposed model. Empirical evaluation on the real SRS dataset, i.e., FAERS, shows that our method can effectively solve the privacy problem in SRS data without influencing the ADR signal strength.

## Background

It is well known that a new drug before hitting the market needs to undergo a series of clinical trials to reveal all possible adverse drug reactions (ADRs). Unfortunately, many serious ADRs cannot be disclosed in the premarketing stage through the limited number of volunteers participate in clinical trials; on the contrary, they can only be identified through long term surveillance of extensive usages of the drug on the masses. Therefore, most of highly developed countries have established various spontaneous reporting systems (SRSs) to collect adverse drug events (ADEs) as a data repository for ADR detection and analysis, e.g., the FDA Adverse Event Reporting System (FAERS) of the US Food and Drug Administration (FDA) [1], the UK Yellow Card scheme [2], and the MedEffect Canada [3], among others.

Usually, the data collected by the SRSs contain sensitive personal health information that should be protected to prevent the identification of individuals. This procures the need of anonymizing the raw data before being published, namely privacy-preserving data publishing (PPDP) [4]. Although in the past few years there have been a lot of researches on this topic, none of the anonymization methods is favorable for SRS datasets, due to which contain some characteristics, including rare events, multiple individual records, and multi-valued sensitive attributes.

In this paper, we present a new privacy-preserving model, called MS(*k*, θ^*^)-bounding, for protecting the published spontaneous ADE reporting data from privacy attacks. We also propose an anonymization algorithm for sanitizing the raw data to meet the requirements specified through the proposed model. Empirical study conducted using FAERS datasets show that our method can effectively prevent the disclosure of patient sensitive information without sacrificing data utility for ADR signal detection. In what follows, we present some background knowledge related to this work, including ADR signal detection and privacy-preserving models, followed by a summarization of our previous work [5] on the deficiency of contemporary PPDP models for publishing SRS datasets.

### Spontaneously reporting systems and ADR signal detection

According to WHO, the definition of ADRs or ADEs is uncomfortable, noxious, unexpected, or potentially harmful reactions resulting from the use of given medications for patients. Usually, an ADR signal (rule) can be represented as an association between symptoms and drugs with some extra conditions, for example, a rule “Avandia, age > 18 years old ⇒ death.”

Statisticians have developed various criteria based on the concept of measuring disproportionality or information component (IC) to evaluate the significance of an ADR signal [6]. The most widely adopted disproportionality-based measurements are Proportional Reporting Ratio (PRR) [7] and Reporting Odds Ratio (ROR) [8]. The PRR measure is used by the U.K. Yellow Card database and UK Medicines and Healthcare products Regulatory Agency (MHRA), while ROR is used by the Netherlands Pharmacovigilance Foundation. All of these measurements can be calculated using a contingency table as shown in Table 1. Table 2 shows some ADR measures and thresholds that commonly used in the pharmacovigilance community for detecting ADR signals.

**Table 1:** The 2 × 2 contingency table used for the identification of ADRs

	Suspected ADR	Without the suspected ADR	Total
Suspected drug	*a*	*b*	*a* + *b*
Other drugs	*c*	*d*	*c* + *d*
Total	*a* + *c*	*b* + *d*	*N* = *a* + *b* + *c* + *d*

**Table 2:** Commonly used ADR measures and thresholds

Measure	Formula	Threshold
PRR	$\frac{a/\left(a+b\right)}{c/\left(c+d\right)}$	PRR − 1.96 × SD > 1
		PRR ≥ 2, *a* ≥ 3, *χ* ^2^ ≥ 4
ROR	$\frac{a/c}{b/d}$	ROR − 1.96 × SD > 1
IC	${ \log}_2\frac{a\left(a+b+c+d\right)}{\left(a+b\right)\left(a+c\right)}$	*Expect*(*IC*) − 1.96 × SD > 0

### Privacy models for microdata publishing

Microdata refer to a kind of data which contains individual information and usually can be represented as tables including tuples defined in a set of attributes, and we can divide these attributes into the following categories:

- *Explicit Identifiers (ID):* These refer to attributes that can uniquely identify each individual, such as SSN, Name, etc.
- *Quasi-identifiers (QID):* These refer to attributes that might be linked with external information to re-identify some of the individuals, e.g., Sex, Age, ZIP code, etc.
- *Sensitive Attributes (SA):* These refer to attributes that contain sensitive information, such as Disease, Salary, etc.
- *Non-sensitive Attributes (NSA):* These refer to attributes not fall into the above three categories.

Since Sweeney [9] pointed out that publishing microdata by only removing *ID* without paying attention to *QID* may threat the privacy of data owners, there have been a lot of researches on this topic [4]. These research efforts towards protecting released microdata aim at thwarting two primary types of privacy attacks, *individual disclosure* and *attribute disclosure*.

Individual disclosure (also known as *table linkage attack*) refers to the situation that a specific tuple for an individual in the published table is re-identified. The most famous privacy model for this purpose is *k*-anonymity [9]. With *k*-anonymity, the data publisher should generalize *QID* of the data such that each *QID* group contains no less than *k* tuples, making a given record indistinguishable from at least *k* - 1 other records by *QID*. Attribute disclosure (also known as *attribute linkage attack*) refers to the situation that the sensitive attribute value of an individual can be inferred without the necessity to link the value to a specific tuple. The prevailing model for this protection is *l*-diversity [10], which requires each *QID* group contains at least *l* “well-represented” sensitive values so as to ensure the probability of inferring the specific sensitive value within each *QID* group will be no more than 1/*l*.

### Problems of contemporary privacy models

We summarize our previous work on the deficiency of contemporary PPDP models for publishing SRS datasets [5]. First, we present the features of SRS data, and then summarize the results of our analysis.

### Special features of SRS data

- *Rare Events:* Usually, undiscovered or new ADRs are rarely observed, so almost all criteria used in measuring the significance of ADRs ignore or overlook the frequency of ADRs. For example, the MHRA measure may output a suspected signal even it occurs only three times. With PPDP models, we often generalize or suppress the records, which may increase the risk of false positive as well as false negative signals of ADRs, especially when we perform stratified ADR detection by factors such as Age, Gender, and Location, i.e., members of the typical *QID*.
- *Multiple Individual Records:* A typical SRS data usually contains reports called follow-ups, which complement the information of an initial report and have to be merged with the initial report to form a more accurate and complete version. Most of contemporary PPDP models assume that there is only one record for each individual, e.g., *k*-anonymity, *l*-diversity. Overlooking the existence of multiple individual records might impair the privacy requirement to be achieved. For example, consider a table satisfying *k*-anonymity. A *QID* group might contain *k* tuples, all of which are of the same individual, thus ruin the privacy requirement.
- *Multi-valued Sensitive and Quasi-sensitive Attribute:* Quasi-sensitive attributes (*QSA*) are not sensitive attributes, but as link to external knowledge may reveal sensitive information of an individual. Typical SRS datasets, e.g., FAERS, usually contain Drug and PT (Preferred Terms of symptoms), each of which, if being linked with external knowledge of clinical treatments, could reveal the disease information of an individual. For example, Prezista and Ritonavir are commonly used together for treating HIV; knowing a patient taking these medicines is almost equivalent to perceiving him having HIV. Besides, FAERS contains another attribute named INDI_PT, which records the indications of the patient before treatment. Values of this attribute can be sensitive (represent some disease, e.g., Multiple Sclerosis) or quasi-sensitive (describe symptoms of some illness, e.g., Muscle Spasticity, possibly caused by Parkinson’s disease). All of these three attributes are multi-valued, i.e., containing more than one value. Very few PPDP models can handle multi-valued sensitive attributes and consider the existence of quasi-sensitive attributes.

### Analysis of previous work

Our previous work in [5] can be summarized as follows:

1. Variants of *k*-anonymity or *l*-diversity overlook the existence of rare instances in the dataset.
2. Only very few models, e.g., (*X*, *Y*)-privacy [11], consider multiple individual records.
3. Except *QS l*-diversity [12], no model notices the existence of quasi-sensitive attributes, not to mention the case of multivalued quasi-sensitive attributes.
4. Most models entail the assumption of single sensitive attribute, while very few embrace the situation of multiple sensitive attributes, e.g., (*α*, *k*)-anonymity [13], Multi-sensitive *l*-diversity [14].
5. No model takes into account all of the mentioned features of SRS datasets, which raises the need to design a new PPDP model to handle these features.

## Methods

### The proposed MS(k, θ^*^)-bounding model

To solve the aforementioned problems, we developed a privacy model called Multi-Sensitive (*k*, θ^*^)-anonymity (abbrev. MS(*k*, θ^*^)-anonymity). Let *D* be SRS data to be published that consists of four disjoint sets of attributes, *QID*, *SA*, *QSA*, and *NSA*, i.e., *D* = <*QID*, *SA*, *QSA*, *NSA*>, and D^*^ the released SRS data after anonymization. We called the records with the same *QID* values a “*QID*-group.” We also assume an external knowledge table *E* about treatment is available, which can be constructed from websites such as Drugs.com, wrongdiagnosis, etc. For simplicity, let *E* contain a pair of attribute group (*Q*_*E*_, *S*_*E*_), where *Q*_*E*_ denotes the set of attributes that can be linked with *QSA* in *D*, e.g., *Drug*, and *S*_*E*_ the set of sensitive attributes, e.g., *Disease*.

**Definition 1 (Confidence).** Let *s* be a sensitive value in *SA* or *S*_*E*_. For a *QID*-group in *D* (or D^*^) with value of *q*, we define the probability that *q* have *s* as

$$ conf\left(q\to s\right), $$

and the same probability after linking *E* via *SA* and *S*_*E*_ as

$$ conf\left(q\to s,E\right). $$

**Definition 2 (Confidence Bounding).** Let *S* = {*s*_1_, *s*_2_, …, *s*_*l*_} be the set of sensitive values to be protected and θ^*^ = (*θ*_1_, *θ*_2_, …, *θ*_*l*_) be the user specified disclosure probability thresholds associated with *S*, where *θ*_*i*_ denotes the threshold for *s*_*i*_, 1 ≤ *i* ≤ *l*. That is, *θ*_*i*_ is an upper bound of the confidence to infer any *QID*-group having *s*_*i*_, with or without external knowledge *E*, i.e.,

$$ conf\left(q\to {s}_i,E\right)\le {\theta}_i. $$

Note that *S* is a subset of all values legal in *SA* and *S*_*E*_, i.e.,

$$ S\subseteq {{\mathrm{U}}_A}_{\in\ SA \cup SE}\ dom(A), $$

where *dom*(*A*) represents the domain of attribute *A*.

Definition 3 (MS(*k*,θ^*^)-bounding). Given *S* and the corresponding θ^*^, we say a release data D^*^ satisfies MS(*k*, θ^*^)-bounding if

1. Every *QID-*group contains at least *k* distinct individuals (cases);
2. The confidence to infer any *QID*-group *q* having *s*_*i*_ is less than *θ*_*i*_, i.e., *conf*(*q* → *s*_*i*_, *E*) ≤ *θ*_*i*_*.*

In MS(*k*, θ^*^)-bounding, we define θ^*^ to control the ratio of sensitive values in *QID* group because not all sensitive values is “really” sensitive. For example, most diseases are sensitive for people, but it does not matter when the others know someone got a flu. This model can solve the multiple individual records problem because *k* is defined by the distinct individuals, and it is easy to check whether the *QID* group satisfies the model or not.

Another noteworthy thing is about the setting of confidence bounding θ^*^. In general, as applying MS(*k*, θ^*^)-bounding to the dataset, every *θ*_*i*_ in θ^*^ should be no less than the frequency of *s*_*i*_ in the dataset, i.e., we must set every *θ*_*i*_ so as to satisfy *θ*_*i*_ ≥ *P*(*s*_*i*_). This is because after generalization the occurrence of *s*_*i*_ in every *QID*-group is no less than *P*(*s*_*i*_), and so setting *θ*_*i*_ < *P*(*s*_*i*_) nullifies the work of anonymization, i.e., the result fails to meet the privacy requirement. However, for a dataset containing some relatively frequent sensitive values, we still can apply MS(*k*, θ^*^)-bounding to the dataset using some other methods like adding counterfeit records or suppressing some of those sensitive values, though those method may severely decrease the utility of the data.

**Example 1.** Table 3 illustrates a sample of the FAERS data, where *ISR* and *CaseID* denote the *ID*s of a record and an event, respectively. Since an event may have more than one reporting records, a CaseID can correspond to many different ISRs. Here we assume *QID* comprises {*Age*, *Gender*, *Country*}. Table 3(a) shows the anonymized table D^*^ composed of two *QID* groups, ([20–30], M, USA) and ([30–40], F, UK), each of which contains two different events; Table 3(b) represents the external table *E* showing knowledge of treating diseases with drugs. It is not hard to derive that the probability of each disease associated with a specific *QID* group is less than 0.4, e.g., *conf*([30–40], F, UK → Headache, *E*) = 0.25. This anonymized table D^*^ thus satisfies MS(2, 0.4)-bounding.

**Table 3:** A sample FAERS data satisfying MS(2, 0.4)-bounding

(a) Anonymized table					
ISR	CaseID	Age	Gender	Country	Drugs
001	001	[20–30]	M	USA	Paracetamol
002	001	[20–30]	M	USA	Paracetamol
003	002	[20–30]	M	USA	Intron A, Antacid
004	002	[20–30]	M	USA	Intron A, Antacid
005	003	[30–40]	F	UK	Paracetamol
006	004	[30–40]	F	UK	Antacid
(b) External table					
Drug	Diseases				
Asprin	Flu, Headache, Fever				
Intron A	Hepatitis B, Hepatitis C, Leukemia, Melanoma				
Paracetamol	Headache, Fever				
Antacid	Stomachache, GERD				

### Anonymization algorithm for MS(*k*, θ^*^)-anonymity

#### Algorithm basics

Our algorithm is a hybrid of greedy and clustering approaches. We view each *QID*-group as a cluster and so develop a clustering-based method [15] to form all *QID*-groups.

Each *QID*-group (cluster) begins with a randomly selected record, and then is gradually increased by adding an isolated record that exhibits the best characteristic among all candidates. This process continues until the group is composed of *k* different cases. Finally, the *QID*s of all records belonging to the same cluster are generalized to the same value.

We use generalization rather than suppression as the anonymization operation because suppression tends to remove records corresponding to rare events. We adopt both *hierarchy-based generalization* and *hierarchy-free generalization*; the former is used when a value generalization hierarchy is defined for the attribute (usually, it is categorical), otherwise the latter is used. For example, we adopt the age hierarchy defined in MeSH [16], a domain knowledge of value generalization hierarchies widely used in medical and healthcare areas. In the following, we describe the metric for evaluating an isolated record quality.

Intuitively, the best record to be included into a *QID*-group should exhibit the most similarity to the group. This implies its addition will result in the least degree of generalization (*distortion of data*, *destruction of utility*, or *information loss*) to be performed on the *QID* attributes of the group. Here in this study, we adapt the measure of information loss defined in [15].

**Definition 4 (Information Loss).** Let *g* denote a group (cluster) constructed during the execution of our algorithm, where the *QID* comprise two different sets, numerical attributes *N*_1_, *N*_2_, …, *N*_*m*_, and categorical attributes *C*_1_, *C*_2_, …, *C*_*n*_, and each *C*_*i*_ is associated with a generalization hierarchy *T*_*i*_. The *information loss* (*IL*) of group *g* is defined as follows:

1 $$ IL(g)=\left|g\right|\times \left({\displaystyle \sum_{i=1}^m\frac{ \max \left({N}_i,g\right)- \min \left({N}_i,g\right)}{ \max \left({N}_i\right)- \min \left({N}_i\right)}+{\displaystyle \sum_{j=1}^n\frac{h\left({C}_j,g\right)}{h\left({C}_j\right)}}}\right) $$

where max (*N*_*i*_) (min(*N*_*i*_)) and max(*N*_*i*_, *g*) (min(*N*_*i*_, g)) denote the maximum (minimum) values of attribute *N*_*i*_ in the whole dataset and group *g*, respectively; |*g*| denotes the number of records in *g*; *h*(*C*_*j*_) is the height of the hierarchy tree *T*_*j*_, and *h*(*C*_*j*_, *g*) the height of the generalized value of *C*_*j*_ for all records in *g*, i.e., the lowest common ancestor in *T*_*j*_ with respect to every *C*_*j*_ value in *g*.

The information loss measures how generalization impact the data utility. As we are building a group *g* by adding new record*s*, we can use the difference of *IL* (Δ*IL*) between the original group and the group with record *r* to determine the best record that produces the least Δ*IL*, i.e.,

2 $$ \Delta IL\left(g,r\right)=IL\left(g \cup \left\{r\right\}\right)\hbox{--}\ IL(g), $$

and the best choice *r*_bst_ is

3 $$ {r}_{\mathrm{bst}} = {\mathrm{argmin}}_r\Delta IL\left(g,r\right). $$

In addition to the data distortion, the privacy requirement is another factor critical for the determination of the best record. This is because the inclusion of a new record would increase the disclosure risk of some sensitive values in the resulting *QID*-group. We introduce a new parameter called *Privacy Risk* (*PR*) to measure the risk of sensitive value disclosure incurred by adding new records into the *QID*-group, thus alleviating the breach of our privacy requirement.

Let *S*_*r*_ denote the set of sensitive values contained in record *r*. Consider a *QID*-group *g* and a sensitive value *s*∈*S*_*r*_. Let *σ*_*s*_(*g*) represent the number of records in *g* containing sensitive value *s*. We define the maximum number of records in *g*, *η*_*s*_(*g*), that will cause the breach of the bound *θ*_*s*_ associated with *s*

4 $$ {\eta}_s(g) = \left\lfloor \max\ \left\{k,\ \left|g\right|\right\}\times {\theta}_s\right\rfloor, $$

and the privacy risk to explore *s* with the inclusion of record *r* as

5 $$ P{R}_s\left(g\cup \left\{r\right\}\right)=\left\{\begin{array}{ll}\frac{\sigma_s(g)}{\eta_s\left(g\cup \left\{r\right\}\right)-{\sigma}_s(g)}\hfill & \mathrm{if}\ {\eta}_s\left(g\cup \left\{r\right\}\right)>{\sigma}_s(g)\hfill \\ {}\infty \hfill & \mathrm{otherwise}\hfill \end{array}\right. $$

Since a record may contain multiple sensitive values, the privacy risk of group *g* caused by including *r* can be defined as the summation of the risk to each sensitive value.

**Definition 5 (Privacy Risk).** Let *g* denote a group (cluster) constructed during the execution of our algorithm. The privacy risk (*PR*) to group *g* caused by including a record *r* is

6 $$ PR\left(g,r\right)=\left\{\begin{array}{ll}1+{\displaystyle \sum_{s\in {S}_r}P{R}_s\left(g\cup \left\{r\right\}\right)}\hfill & \mathrm{if}\ {\eta}_s\left(g\cup \left\{r\right\}\right)>{\sigma}_s(g)\hfill \\ {}\infty \hfill & \mathrm{otherwise}\hfill \end{array}\right. $$

Finally, we refine Δ*IL* into Δ*IL’* as follows:

7 $$ \Delta IL'\left(g,\ r\right) = \Delta IL\left(g,r\right)\times PR\left(g,r\right), $$

and

8 $$ {r}_{\mathrm{bst}} = {\mathrm{argmin}}_r\Delta IL'\left(g,r\right). $$

Note that when all sensitive values in *S*_*r*_ are new to group *g*, *σ*_*s*_(*g*) = 0 and so is *PR*(*g*, *r*), which will dismiss the effect contributed by information loss (Δ*IL*). To avoid this situation, we add an increment into (6).

**Fig. 1:**
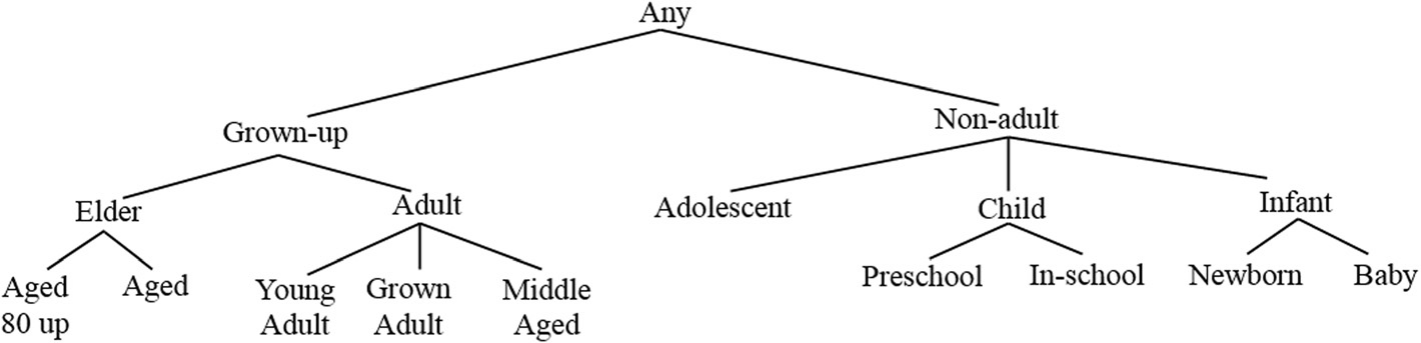
Value hierarchy for age

**Fig. 2:**
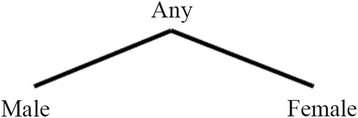
Value hierarchy for gender

**Example 2.** Consider Table 4 which consists of a group of four cases, r1 to r4, with *g* = {Male, Young Adult, 50–75} and three isolated cases, r5 to r7. Figure 1 shows the age hierarchy defined in MeSH and Fig. 2 depicts a simple hierarchy for gender. Let *QID* = {*Gender*, *Age*, *Weight*} and *SA* = {*Indications*}, and suppose *k* = 5, θ^*^ = 0.6, and weight range = 0 ~ 100. The information loss for group *g* is

**Table 4:** An example data

(a) A known group *g*				
CaseID	Gender	Age	Weight	Indications
r1	Male	Young Adult	[50–75]	I1
r2	Male	Young Adult	[50–75]	I2, I3, I4
r3	Male	Young Adult	[50–75]	I2, I3
r4	Male	Young Adult	[50–75]	I2
(b) Isolated records				
CaseID	Gender	Age	Weight	Indications
r5	Male	Adocent	50	I1
r6	Female	Adult	40	I3, I4
r7	Male	Young Adult	80	I2, I3

$$ IL(g)=4\times \left(\frac{0}{1}+\frac{0}{2}+\frac{75-50}{100-0}\right)=4\times 0.25=1 $$,

and for *g* ∪ {r5}, *g* ∪ {r6}, and *g* ∪ {r7} the values are

$$ IL\left(g\cup \left\{r5\right\}\right)=5\times \left(\frac{0}{1}+\frac{2}{2}+\frac{75-50}{100-0}\right)=5\times \left(1+0.25\right)=6.25 $$

$$ IL\left(g\cup \left\{r6\right\}\right)=5\times \left(\frac{1}{1}+\frac{1}{2}+\frac{75-40}{100-0}\right)=9.25 $$

$$ IL\left(g\cup \left\{r7\right\}\right)=5\times \left(\frac{0}{1}+\frac{0}{2}+\frac{85-50}{100-0}\right)=5\times (0.35)=1.75 $$

Next, it is easy to compute the Δ*IL*s.

$$ \Delta IL\left(g,\ \mathrm{r}5\right) = 6.25\ \hbox{--}\ 1.4 = 4.85 $$

$$ \Delta IL\left(g,\ \mathrm{r}6\right) = 7.25\ \hbox{--}\ 1.4 = 5.85 $$

$$ \Delta IL\left(g,\ \mathrm{r}7\right) = 1.75\ \hbox{--}\ 1.4 = 0.35 $$

The privacy risks are

$$ PR\left(g,\mathrm{r}5\right)=1+\frac{1}{3-1}=1.5 $$

$$ PR\left(g,\mathrm{r}6\right)=1+\frac{2}{3-2}+\frac{1}{3-1}=3.5 $$

$$ PR\left(g,\mathrm{r}7\right)=\infty $$

Finally, we can compute the Δ*IL’*s and obtain the best choice *r*_bst_ among r5 to r7, concluding *r*_bst_ = r5.

$$ \begin{array}{l}{r}_{\mathrm{bst}} = {\mathrm{argmin}}_r\left\{\Delta IL'\left(g,\ \mathrm{r}5\right),\ \Delta IL'\left(g,\ \mathrm{r}6\right),\ \Delta IL'\left(g,\ \mathrm{r}7\right)\right\}\\ {}\kern1.3em  = {\mathrm{argmin}}_r\left\{4.18\times 1.5,\ 5.85\times 3.5,\infty \right\} = \mathrm{r}5\end{array} $$

#### Detail description

Algorithms 1 and 2 present the description of our algorithm, which is composed of two stages. The first stage is to create as many *QID*-groups that satisfy MS(*k*, θ^*^)-bounding as possible. We introduce a concept called *combined record* (or *super record*) to handle the issue of multiple individual records. That is, all records with the same CaseID are combined into a super record before the anonymization procedure. This avoids the abnormal situation that members of this CaseID group will be, after generalization, divided into different *QID*-groups, which will cause larger bias on the data quality and perplex the process of identifying duplicate records during ADR signal detection.



Initially, we combine records with the same CaseID into one super record per CaseID by generalizing the values of those records. The generalization is necessary because not all members of the same CaseID exhibit the same *QID* value. This is due to the existence of follow-up records, which represent compensation for the initial report and so may contain update information.



Next, we create an empty group and add into it a randomly selected record, then into which we add more records step by step, each with the least Δ*IL’* (defined in (7)) until the group satisfies MS(*k*, θ^*^)-bounding. Thirdly, we choose a new record that is most distinguished from the one chosen for creating the latest group and repeat the same steps to grow the group. These steps are repeated until the remaining records cannot form a group, e.g., the number of records is less than *k* or most of the remaining records contain the same sensitive value.

The second stage is then activated by calling function *QID*-generalization (see Algorithm 2). First, we take care of the ungrouping records by adding each of them into the group that produces the least Δ*IL*’ to ensure the utility and meet the privacy requirement. Next, we split those combined records back to their original records (do not change the group they belong to). Finally, we generalize all records within the same group into the same *QID*s such that the whole data set will satisfy MS(*k*, θ^*^)-bounding.

## Results and Discussions

We have conducted a series of experiments to confirm if our model is more suitable for anonymizing SRS datasets than prevailing PPDP models. We describe the design of each experiment, present the experimental results, and state our observations.

### Experimental design

All experiments were conducted over FAERS datasets, which is a SRS system provided by U.S. Food and Drug Administration (FDA) and released quarterly. Each report in FAERS is uniquely identified by an attribute called *ISR*, and contains an attribute *CaseID* to identify distinct individuals, along with some demographic information such as *Weight*, *Age*, and *Gender*, drugs information such as drug name (*Drug*) and indication (*INDI_PT*), and reaction information (*PT*).

We used {*Weight*, *Age*, *Gender*} as *QID*, *CaseID* as the individual identifier, and used drug indication (*INDI_PT*) and drug reaction (*PT*) as *SA*. Datasets from 2004Q1 to 2011Q4 were selected to build the test sets, where any record with *QID* containing missing values was discarded.

Four prevailing PPDP models were evaluated against our model. They are *k*-anonymity, (*X*, *Y*)-anonymity, Multi-sensitive *l*-diversity, and (*α*, *k*)-anonymity. These models were chosen because each of them is the representative or the prevailing models, and can be applied to anonymize SRS data without additional modification; this is why *l*-diversity is replaced by Multi-sensitive *l*-diversity.

All models were evaluated from two aspects: the quality of resulting anonymized dataset, measured by two criteria, i.e., data utility and privacy risk, and the influence to ADR signals.

For the anonymization quality, we considered two measurements. The first one called *Normalized Information Loss* (*NIL*) is used to measure the data utility, defined as follows:

9 $$ NIL\left({D}^{*}\right)=\frac{1}{n_g\times \left|QID\right|}\left({\displaystyle \sum_{g\in {D}^{*}}IL}(g)\right) $$

where D^*^ is the anonymized data table, *n*_*g*_ denotes the number of *QID*-groups in D^*^, *g* denotes a *QID*-group, and |*QID|* the cardinality of *QID*. The value of *NIL* ranges over [0, 1]; larger *NIL* means poorer data utility.

The second one called *Dangerous Ratio* (*DR*) is used to measure the privacy risk of anonymized dataset, defined as follows:

10 $$ DR=\frac{\mathrm{number}\ \mathrm{of}\ \mathrm{dangerous}\ QID-\mathrm{groups}\ }{\mathrm{number}\ \mathrm{of}\ QID-\mathrm{groups}} $$

A *QID*-group is dangerous if it contains at least one unsafe sensitive value, that is, the attacker’s confidence for inferring that value is higher than the specified threshold. In this sense, the *DR* measure also estimates the privacy-preserving quality of an anonymized table.

For the influence to ADR signals, we inspect the impact of anonymized data on the strength of observed ADR rules. Following our previous work in [17], we chosen from FDA MedWatch [18] all significant ADR rules that render withdrawal or warning of the drugs and associated with patient demographics, such as age or gender conditions. Detail description of these ADR rules is shown in Table 5.

**Table 5:** Selected ADR rules from FDA MedWatch

Drug name	Adverse reaction	Demographic condition	Marked year	Withdrawn or warning year
AVANDIA	Myocardial infarction	18~	1999	2010
	Death			
	Cerebrovascular accident			
TYSABRI	Progressive multifocal leukoencephalopathy	18~	2004	2005
ZELNORM	Cerebrovascular accident	Female	2002	2007
WARFARIN	Myocardial infarction	60~	1940	2014
REVATIO	Death	~18	2008	2014

Since our model allows non-uniform settings of confidence bounding, i.e., θ^*^, we considered three different scenarios of thresholds for θ^*^ to inspect the effect of different settings: 1) Uniform setting for θ^*^, i.e., all confidences of symptoms were set to the same value (0.2 or 0.4); 2) Level-wise specification, that is, all symptoms (or diseases) were classified into three levels, high sensitive, low sensitive, and non-sensitive. Those symptoms corresponding to high sensitive are assigned a smaller threshold, i.e., 0.2, low sensitive are assigned a larger threshold, i.e., 0.4, and non-sensitive are assigned to 1; 3) Frequency-based strategy, the threshold of each symptom is determined based on the idea: “The more frequently the symptom occurs, the less sensitive it is.”

### Results for uniform confidence setting

**Fig. 3:**
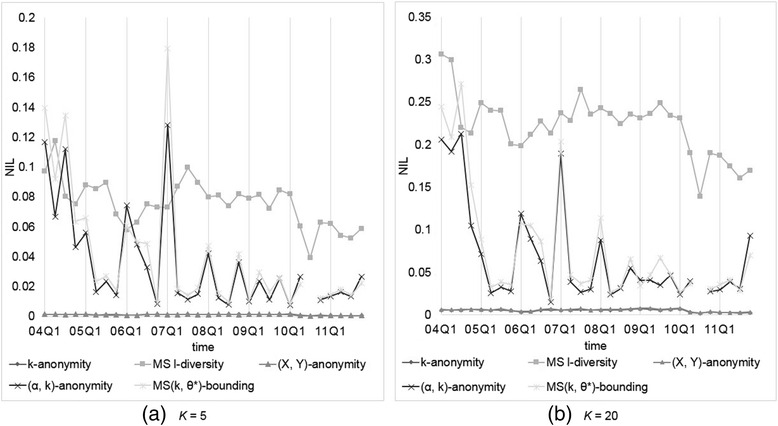
Comparison of models on *NIL*s with θ* (or *α*) = 0.2 and *k* (or *l*) = 5 or 20

**Fig. 4:**
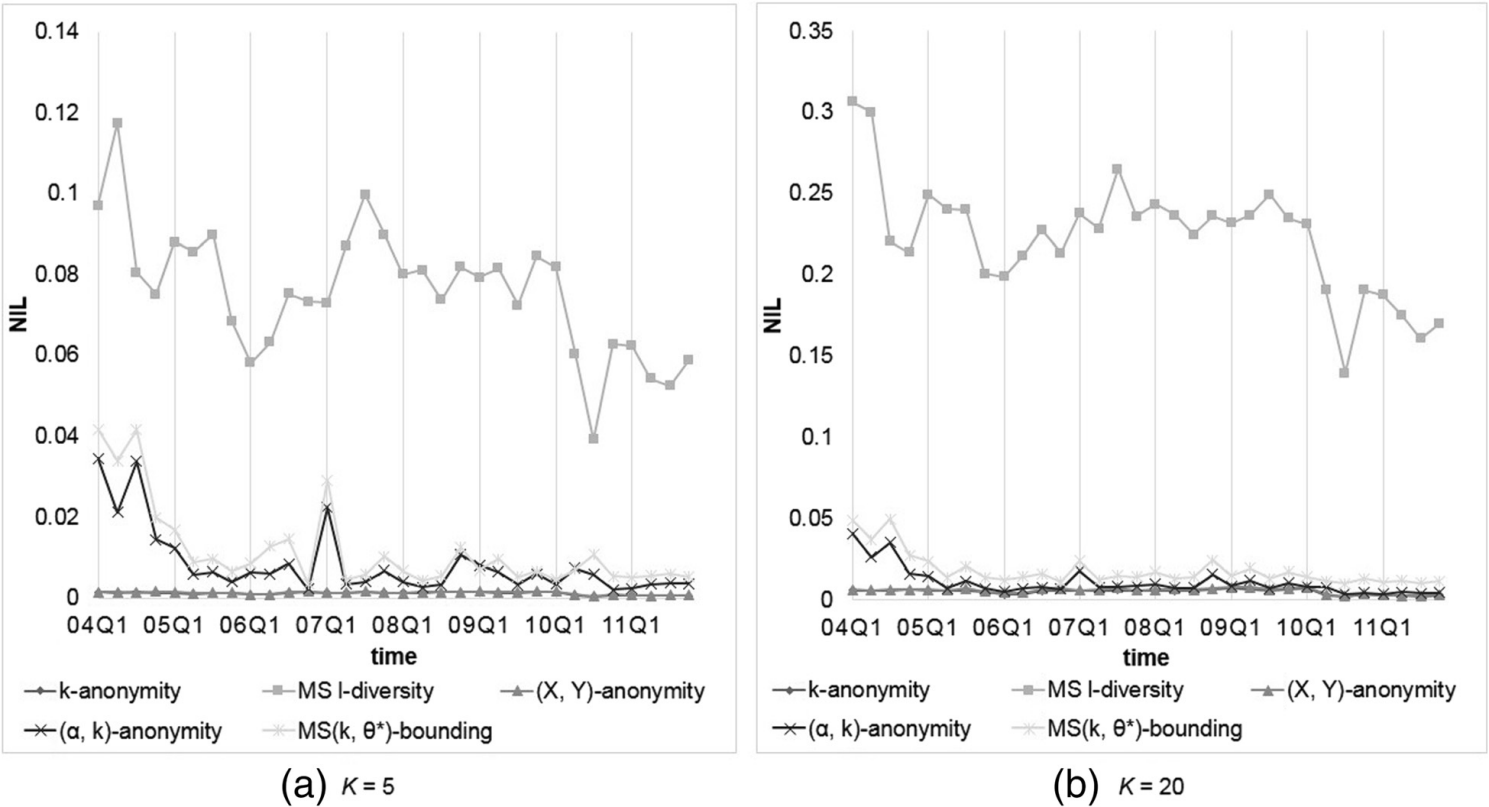
Comparison of models on *NIL*s with θ* (or *α*) = 0.4 and *k* (or *l*) = 5 or 20

We assume every symptom is of the same sensitivity with confidence bounded by 0.2 or 0.4. Analogously, *α* is set to 0.2 or 0.4 for (*α*, *k*)-anonymity, while *k* (or *l*) = 5, 10, 15, 20 for *k*-anonymity, (*X*, *Y*)-anonymity, Multi-sensitive *l*-diversity, and our MS(*k*, θ^*^)-bounding. First, we compared the data utility generated by each anonymization method. Figures 3 and 4 show the resulting *NIL*s for every method, where MS *l*-diversity means Multi-sensitive *l*-diversity. Panels inside are for better view of data position, and applicable to all following figures. Since the results for *k* (or *l*) = 10, 15 are somewhere in between those for *k* (or *l*) = 5, 20 and conform to the overall trend, hereafter we omit these two cases. From the obtained results, we observe that

1. *k*-anonymity and (*X*, *Y*)-anonymity are good at preserving the data utility, and both exhibit nearly identical results, which are less than those generated by our methods.
2. (*α*, *k*)-anonymity and our model yield similar *NIL* results, because under uniform setting the only difference between (*α*, *k*)-anonymity and our model is that (*α*, *k*)-anonymity does not consider duplicate reports, yielding not too much effect in information loss. Furthermore, (*α*, *k*)-anonymity and our model suffer from much less information loss when the confidence threshold is set relatively higher (0.4 vs 0.2).
3. Multi-sensitive *l*-diversity causes much more information loss than the other models because the top-down method tends to create larger *QID*-groups than that by bottom-up method.
4. Even in larger threshold setting, the information loss generated by our method is around 5 to 40 times of that by *k*-anonymity and (*X*, *Y*)-anonymity, though the values are still small, normally between 0.01 to 0.2; the larger *k* value is, so is *NIL*. That it, the data utility decreases as larger *QID*-group is allowed.

It is noteworthy that some datasets anonymized by our method with lower *θ* produce very high *NIL*s, i.e., 2004Q4, 2007Q1, and 2010Q3. After further inspection we found that it is because most of these datasets contain some relatively high frequent symptoms. For example, there are 22,730 reports (without missing values) in 2007Q1, and 3,890 (17.1 %) of them recorded “Diabetes Mellitus Non-Insulin-Dependent,” and in 2010Q3, 12,833 of 63,838 (20.1 %) reports containing “Smoking Cessation Therapy.” In this situation, it is hardly to apply (*α*, *k*)-anonymity or our MS(*k*, θ^*^)-bounding with *α* (so as *θ*) = 0.2 (<20.1 %) to this dataset. It looks like uniform threshold setting of our model is not suitable to data with high frequent sensitive values, but in most scenarios, the more frequent the values occur, the lesser sensitive they are. All we need is to adopt non-uniform setting for θ^*^, as to be shown later.

**Fig. 5:**
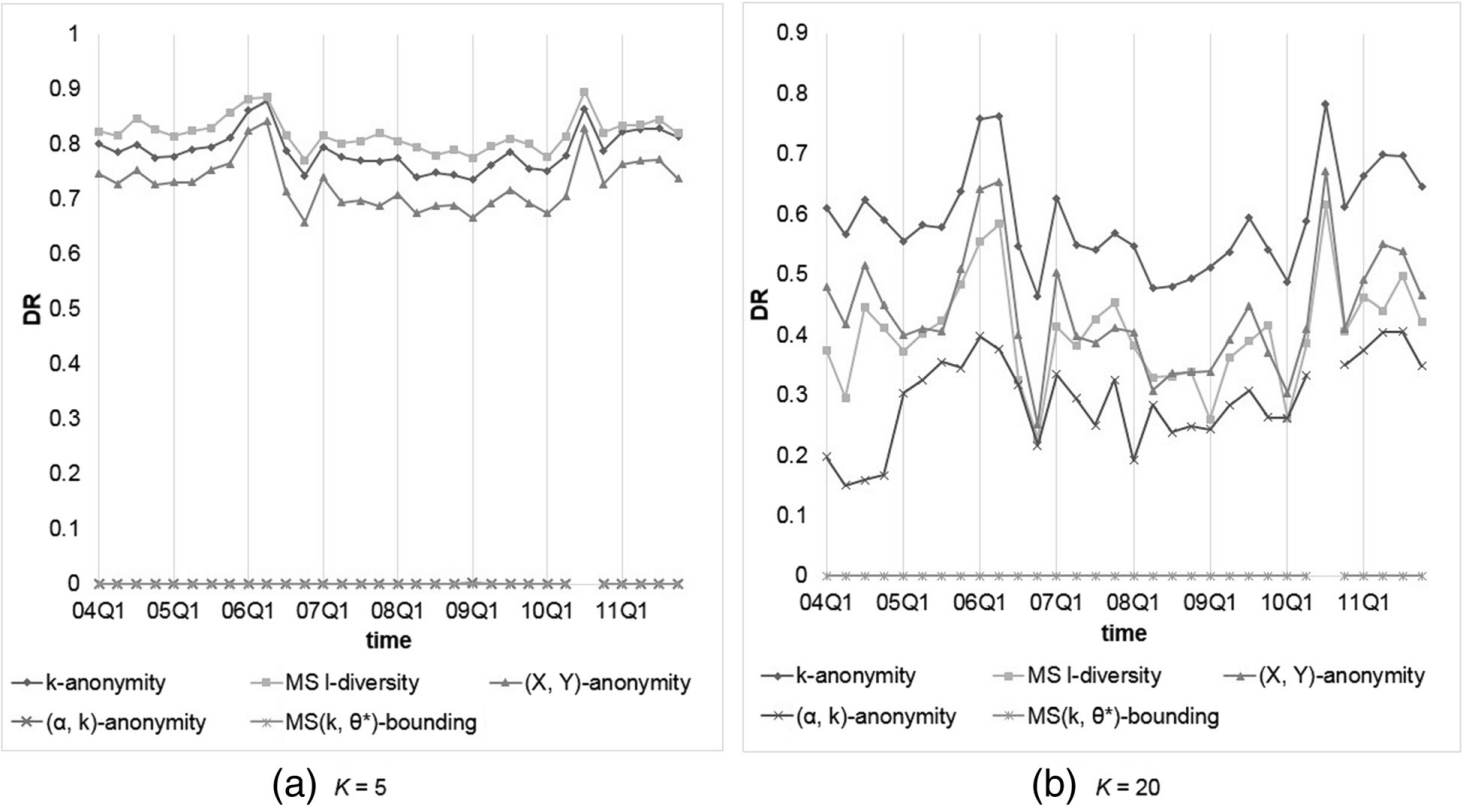
Comparison of models on *DR*s with θ* (or *α*) = 0.2 and *k* (or *l*) = 5 or 20

**Fig. 6:**
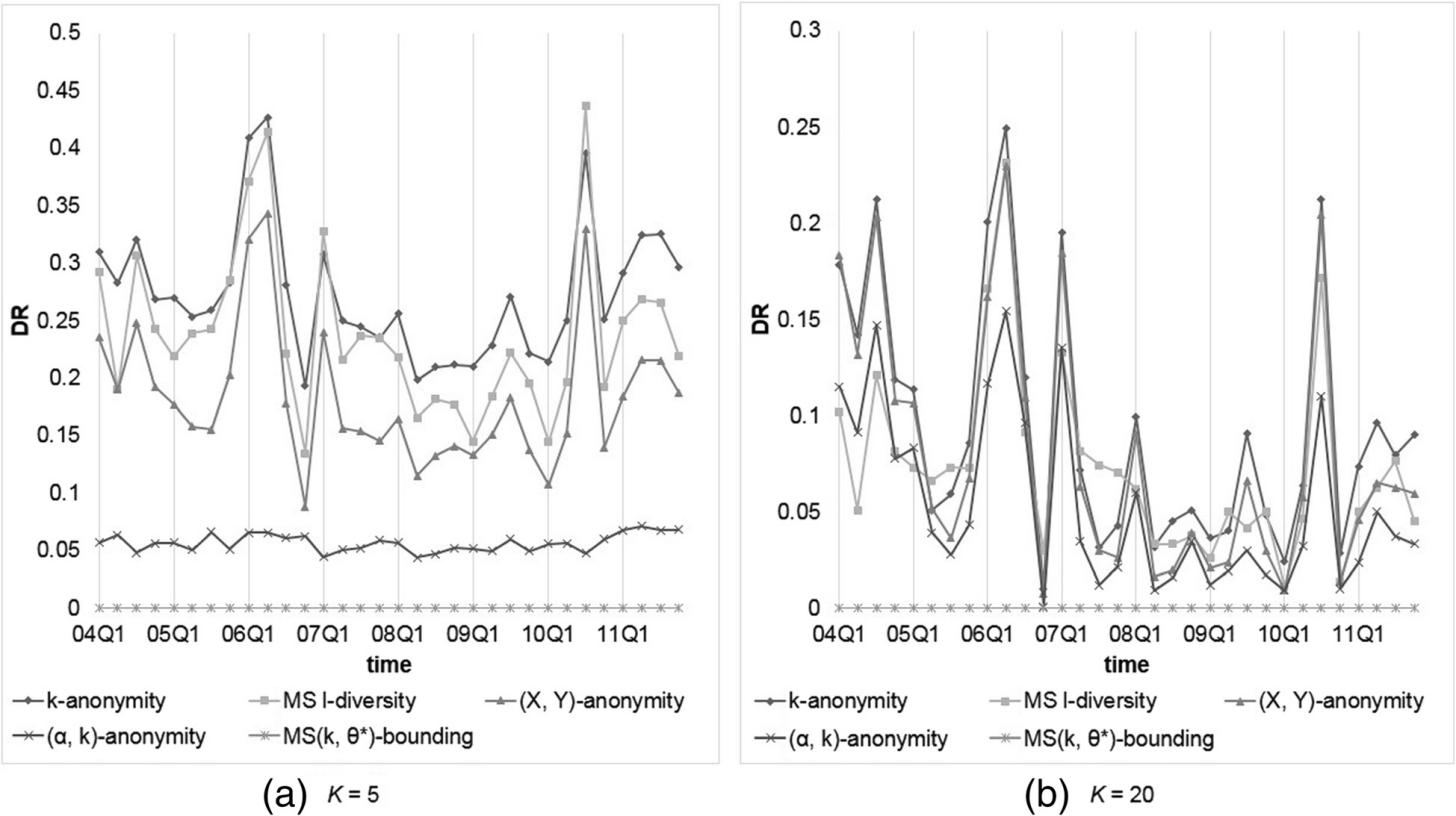
Comparison of models on *DR*s with θ* (or *α*) = 0.4 and *k* (or *l*) = 5 or 20

Next, we compared the privacy risk raised by each anonymization method. Figures 5 and 6 depict the resulting *DR*s for all methods. From the obtained results, we observe that

1. Our MS(*k*, θ^*^)-bounding yields no *DR* because the flexibility to set θ^*^ according to user requirement. On the contrary, (*α*, *k*)-anonymity would suffer from some *DRs* because *QID*-groups contain duplicate reports, which may decrease actual group size, causing violation of the privacy requirement. While *k* is getting larger, the probability of duplicate reports accumulated to the same group is increasing, further aggravating *DR*s.
2. Multi-sensitive *l*-diversity does not perform well on protecting the sensitive values. This is because it only guarantees the number of records with distinct sensitive values in each group no less than *l*, which may fail to thwart the attacker’s confidence on inferring the patient symptoms.

For those models not considering confidence threshold on sensitive values, including *k*-anonymity and (*X*, *Y*)-anonymity, it can be observed that the larger *k* is, the lower *DR* being generated. That it, the data privacy risk increases as larger *QID*-group is allowed.

### Results for level-wise confidence setting

To inspect the applicability of our model to more practical situation, we also adopted level-wise setting of θ^*^. In practice, most symptoms or indications are not “really” sensitive. We choose group of symptoms called “Acquired immunodeficiency syndromes” (a High Level Term (HLT) in MedDRA), which contains 32 PTs and most of them are similar to AIDS, as “high sensitive” with confidence threshold = 0.2, and another two groups called “Coughing and associated symptoms” and “Allergies to foods, food additives, drugs and other chemicals,” which contain 44 PTs, as “non-sensitive” symptoms with confidence threshold = 1. The confidence thresholds of symptoms not belonging to the above groups are set to 0.4.

**Fig. 7:**
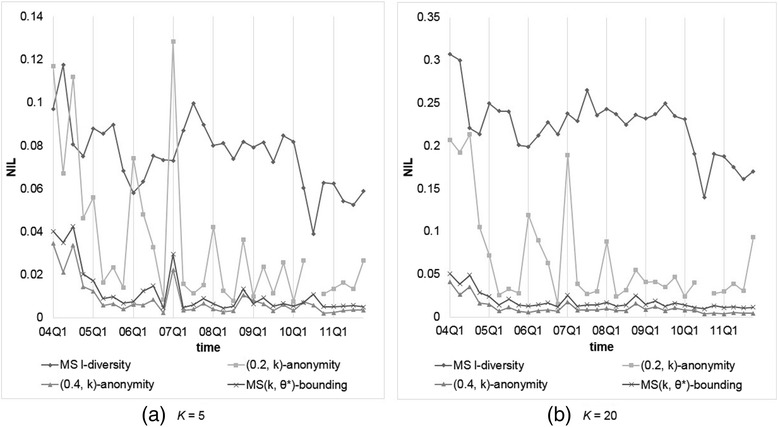
Comparison of our MS(*k*, θ*)-bounding with level-wise setting with other models on *NIL*s with *k* (or *l*) = 5 or 20

**Fig. 8:**
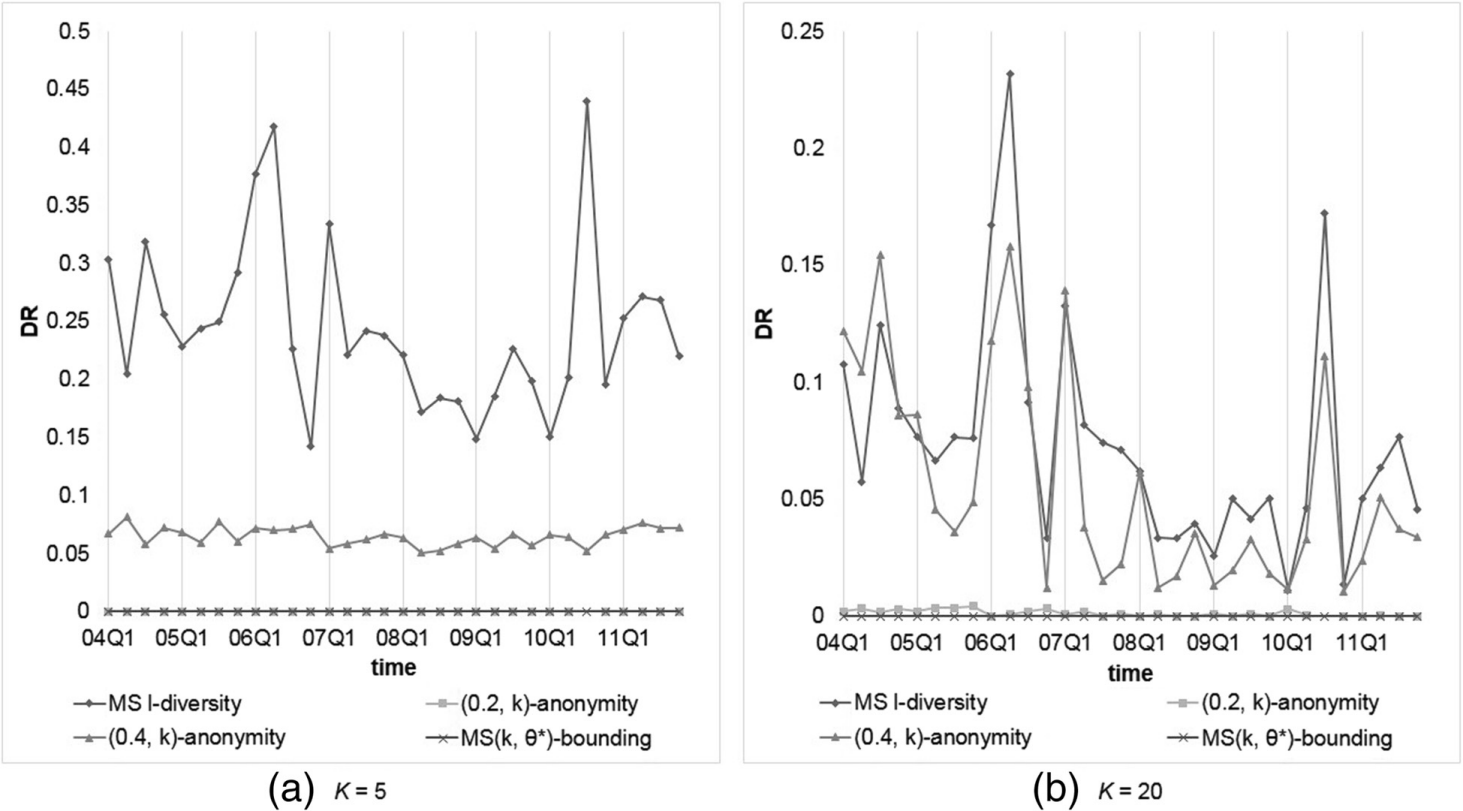
Comparison of our MS(*k*, θ*)-bounding with level-wise setting with other models on *DR*s with *k* (or *l*) = 5 or 20

We compared our MS(*k*, θ^*^)-bounding with those models considering sensitive values, including Multi-sensitive *l*-diversity and (*α*, *k*)-anonymity. The parameter setting is *α* = 0.2 and 0.4, and *k* (or *l*) = 5, 10, 15, and 20. Figures 7 and 8 show the resulting *NIL*s and *DR*s, respectively. From the obtained results, we observe that

1. All *NIL*s generated except by MS(*k*, θ^*^)-bounding are the same as observed previously, but *DR*s are different because of various threshold settings.
2. Because records containing “high sensitive” and duplicate records are rare, (0.2, *k*)-anonymity generate very few *DRs*. However, the generated *NIL* is very high for data with high frequent sensitive value that may decrease data utility severely.
3. MS(*k*, θ^*^)-bounding produces only a little larger *NIL* than (0.4, *k*)-anonymity because in this level-wise specification, most symptoms receive confidence threshold at 0.4. In contrast, MS(*k*, θ^*^)-bounding does not produce any *DR* but (0.4, *k*)-anonymity violates the privacy requirement more often due to overlooking duplicate records.

In summary, the performance of our MS(*k*, θ^*^)-bounding is better than the other models, while Multi-sensitive *l*-diversity yields the worst performance.

### Results for frequency-based confidence setting

Finally, we consider another scenario of confidence setting: the threshold of a symptom is set according to its frequency in the dataset. We calculated the frequencies of all symptoms appear in the dataset and set the confidence thresholds of the most 10 % frequent symptoms to 1, the last 10 % frequent symptoms to 0.2, and the remaining to 0.4, respectively.

**Fig. 9:**
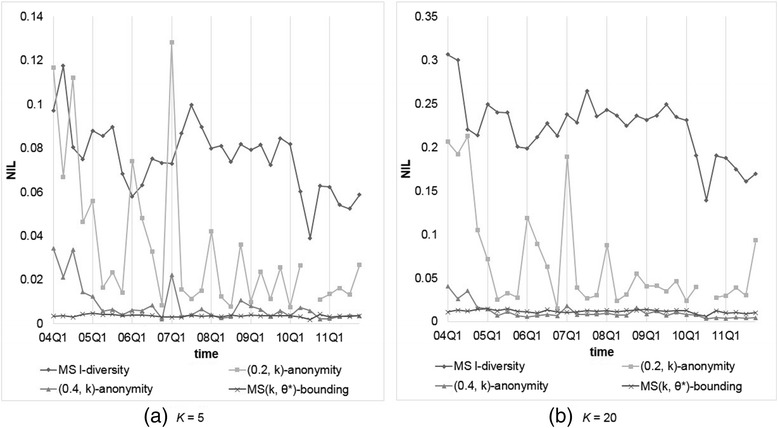
Comparison of our MS(*k*, θ*)-bounding with frequency-based setting with other models on *NIL*s with *k* (or *l*) = 5 or 20

**Fig. 10:**
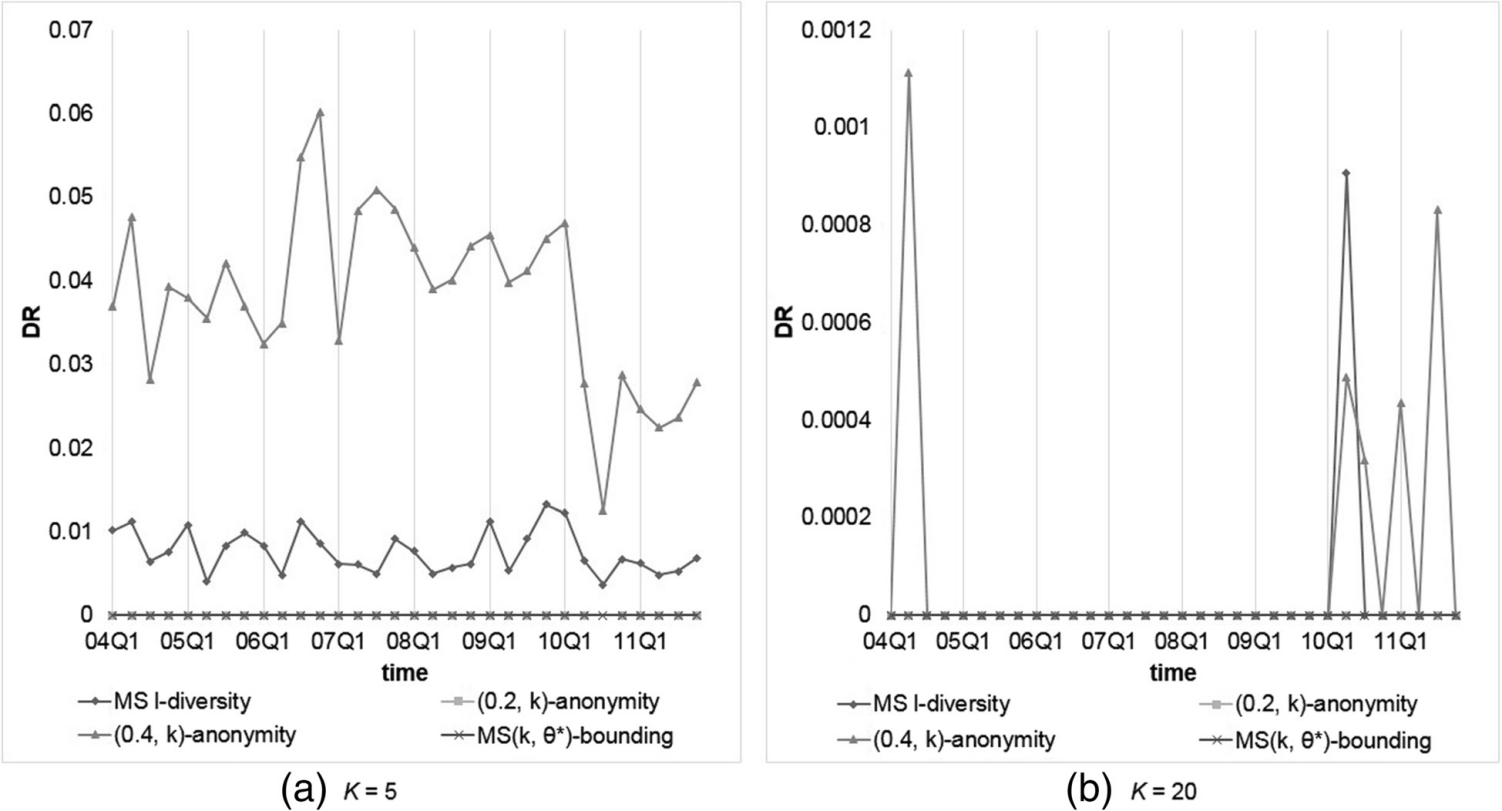
Comparison of our MS(*k*, θ*)-bounding with frequency-based setting with other models on *DR*s with *k* (or *l*) = 5 or 20

Again, we compared our MS(*k*, θ^*^)-bounding with Multi-sensitive *l*-diversity and (*α*, *k*)-anonymity with the same parameter settings, i.e., *α* = 0.2 and 0.4, and *k* (or *l*) = 5, 10, 15, and 20. Figures 9 and 10 show the resulting *NIL*s and *DR*s, respectively. From the obtained results, we observe that

1. As mentioned previously, all *NIL*s generated except by MS(*k*, θ^*^)-bounding are the same as the uniform setting, while *DR*s are different because of different threshold settings.
2. All models generate less *DR*s than that by level-wise setting, because most dangerous groups appearing in the previous experiments are caused by high frequent symptoms, whose thresholds are set to 1 in this experiment.
3. Even 90 % of *θ*'s in θ^*^ are set to 0.4 or lower, our MS(*k*, θ^*^)-bounding produces very small *NIL* than (0.4, *k*)-anonymity when being applied to data with high frequent symptoms such as 2004Q1 and 2007Q1.

In FAERS data, there are more than 20,000 different symptoms, which will require much researching effort and background knowledge to determine the threshold of each symptom. The frequency-based approach is a simple but reasonable method, and with this threshold definition, our MS(*k*, θ^*^)-bounding exhibits the best data utility and the least privacy risk among all the models we examined.

### Influence on ADR signals

We also conducted an experiment to inspect the impact of anonymized data on the strengths of discovered ADR rules. For each ADR rule shown in Table 5, we computed and checked the difference on the number of events, PRR and ROR measures between the original datasets and anonymized datasets. Since all rules exhibit similar phenomenon, we only show the results of the following rule

AVANDIA, age > 18 ⇒ CEREBROVASCULAR ACCIDENT.

**Fig. 11:**
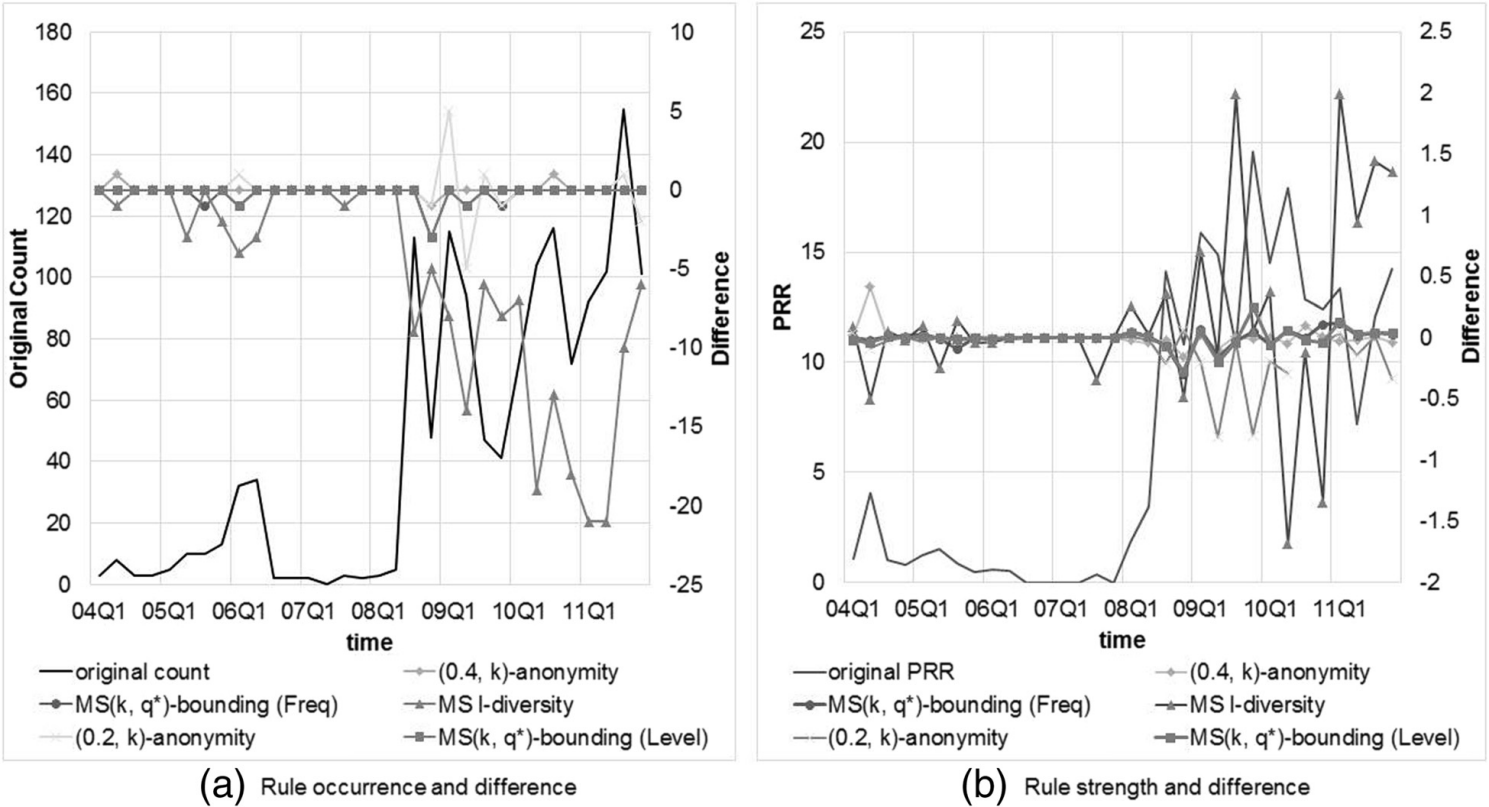
Comparison of rule occurrence and strength (in PRR) generated from original dataset and anonymized dataset by different methods

Figure 11 depicts the occurrence and strength of the above rule in the original dataset (original count and original PRR) and from which the difference yielded from the dataset anonymized by Multi-sensitive *l*-diversity, (0.2, *k*)-anonymity, (0.4, *k*)-anonymity, and our MS(*k*, θ^*^)-bounding with frequency-based setting, with *k* = 20. The obtained results show that

1. Multi-sensitive *l*-diversity does not perform well because of the top-down strategy, which is less flexible to create *QID*-groups.
2. Most of the time there is no difference between the original and anonymized datasets except Multi-sensitive *l*-diversity. All of them are less than five, and the extreme value only occurs when original count is large (more than 80).
3. Not surprisingly, only very small difference on PRR ranging from −1 to 1 were observed from the anonymized datasets (except Multi-sensitive *l*-diversity), which nearly can be ignored.

These observations reveal that our method can effectively solve the privacy problem in SRS datasets without overlooking rare events and influencing the ADR signal strength.

## Conclusions

In this paper, we proposed a new PPDP model for protecting SRS data that possess some characteristics overlooked by contemporary models, including rare events, multiple individual records, and multi-valued sensitive attributes. We also presented an anonymization algorithm to sanitize SRS data in accordance with the proposed model. Empirical studies showed that our method can prevent the disclosure of personal sensitive information without sacrificing the data utility and biasing the discovered ADR signals.

Although our approach is designed mainly for SRS data, it can also be applied to other types of medical data or applications with features analogous to SRS data; for example, electronic health records (EHRs), which contain more detailed private information and so deserve further investigation.

We also notice that FAERS data contain lots of missing values. Existing PPDP methods usually ignore the presence of missing values, simply deleting them before executing data anonymization. However, for data with enormous missing values, like SRS data, deleting all records with missing values may ruin the data utility seriously, so how to deal with missing values is an interesting issue. Another important and challenging issue goes to continuous data publishing [11, 19]. Typically, SRS data are released sequentially. Combining related releases would sharpen the identification of an individual record or sensitive information. We are endeavoring to extend our current approach to solve these problems.
